# Nature does it better: mimicking *in vivo* conditions to resolve *in vitro* production side effects

**DOI:** 10.3389/fvets.2025.1617740

**Published:** 2025-09-25

**Authors:** Enrico Dalle Palle, Koray Tekin, Calogero Stelletta

**Affiliations:** ^1^Department of Animal Medicine, Production and Health, University of Padua, Legnaro, Italy; ^2^Department of Reproduction and Artificial Insemination, Faculty of Veterinary Medicine, Ankara University, Ankara, Türkiye

**Keywords:** oviductal fluid, *in vitro production* IVP, large offspring syndrome, embryos, extracellular vesicles, 3D cell culture

## Abstract

Oviduct represents the original place of fertilisation and early embryo development in all the domestic animals. In past time it has been considered a mere channel but new reproductive biotechnology approaches suggested the need of structurally and functionally efficient oviductal environment for *in vitro* embryo production. Recreating the oviductal microenvironment in IVP systems represents a paradigm shift in reproductive biotechnology. By incorporating reproductive fluids and utilising advanced 3D culture models could be reduced adverse IVP outcomes, and bring assisted reproduction closer to its natural counterpart.

## The oviduct: more than a mere channel

*In vitro* embryo production (IVP) technologies often fail to fully replicate the highly dynamic and selective environment of the oviduct, leading to suboptimal outcomes such as reduced embryo quality and developmental anomalies. Can mimicking the oviductal microenvironment resolve these limitations and improve success rates? To answer this question, it is essential to first understand the complex physiological and anatomical characteristics of the oviduct that support natural fertilization and early embryonic development. The oviduct, also known as the salpinx or Fallopian tube in humans, is a highly specialized reproductive organ essential for fertilization and early embryo development. Previously considered a simple conduit for gamete transport, it is now recognized for its crucial roles in sperm selection, capacitation, fertilization, and early embryonic support (1–3).

Anatomically, the oviduct consists of distinct regions. The uterine-tubal junction (UTJ) acts as a selective barrier, allowing capacitated spermatozoa with specific surface markers, such as members of ADAMs (a disintegrin and metalloprotease) protein family, particularly ADAM3 to pass through, thereby ensuring optimal fertilization to occur (4–7). The isthmus serves as a sperm reservoir and a site for capacitation. The ampulla is the primary site of fertilization, where gamete fusion occurs. The infundibulum captures the ovulated oocyte via fimbriae and directs it into the oviduct. These regions exhibit specialized structures that optimize reproductive processes. The isthmus’s narrow, convoluted structure facilitates sperm storage, while the ampulla’s wider lumen and folded mucosa create an ideal environment for fertilization and early embryonic development (1, 2). These region-specific adaptations raise the question: what if such *in vivo* conditions could be replicated *in vitro* to improve embryo quality?

This mini-review aims to summarize the key structural and functional features of the oviduct, highlight the limitations and pathological consequences of current IVP protocols, and discuss emerging strategies that aim to mimic *in vivo* conditions through the integration of reproductive fluids (RFs), extracellular vesicles (EVs), and three dimensional (3D) culture systems. The reviewed literature includes studies conducted on humans, livestock species (primarily bovine and porcine), and laboratory animals (notably mice and rabbits). The relevance and limitations of animal models in representing human reproductive processes are also briefly addressed.

### Oviductal cells and their functions

The oviductal epithelium consists of ciliated cells and secretory cells. Ciliated cells play a pivotal role in gamete transport by generating directional flow through rhythmic ciliary beating. This motility is tightly regulated by hormonal cues: estrogen significantly enhances ciliary beat frequency and promotes ciliogenesis, while progesterone downregulates this activity during the luteal phase (8). In parallel, estrogen also stimulates the proliferation and activity of secretory cells, increasing the synthesis and release of oviductal fluid (OF), which provides biochemical support for sperm capacitation, fertilization, and early embryo development. The composition of OF dynamically changes in response to ovarian signals, mainly steroids such as 17 beta-estradiol (E2) and progesterone (P4), and uterine signals; in fact the vascular supply of the oviduct, via ovarian and uterine arteries, ensures communication between oviduct, ovary, and uterus through exchanging of metabolites, hormones and signaling molecules, modulating the reproductive microenvironment (9, 10).

### Oviductal fluid: a crucial medium for gametes and embryo

Oviductal fluid is a complex biochemical medium containing nutrients, enzymes, hormones, and signaling molecules (3, 11). Its movement is governed by the activity of the secretory cells, predominantly in the isthmus, ciliary beating, and oviductal peristalsis. These movements are not only mechanical but also under precise hormonal regulation. The release of oviductal fluid is hormonally regulated estrogen enhances the activity of secretory and ciliated cells, promoting fluid production and ciliary beating, while progesterone generally downregulates secretion during the luteal phase. Estrogen and progesterone influence oviductal peristalsis and fluid dynamics, which in turn facilitate gamete interaction and synchronize embryo transport with uterine implantation window (4, 12–16). Prior to fertilization, OF undergoes key modifications that facilitate gamete interaction and fertilization. Changes in viscosity regulate sperm motility, while the release of chemotactic agents guides sperm toward the oocyte. Viscosity greatly influences the chemotaxis of sperm in the oviduct by altering their motility and facilitating navigation through viscous fluids. Sperm exhibit increased linear and progressive movements in high-viscous fluid compared to the low viscosity environments. The interplay between change of viscosity, flow (rheotaxis) and temperature (thermotaxis) seem to enhance the energetic efficiency sperm motility (3). Thermotaxis, which relies on temperature gradients, aids sperm migration to the ampulla (9). Polyspermy is regulated through zona pellucida modifications (cortical/zona reaction) and the release of repellent-like molecules (i.e., osteopontin) immediately after fertilization by the oviduct. Emerging studies highlight the impact of OF on epigenetic modulation, potentially mitigating IVP-associated anomalies (1, 17). Addressing these challenges requires media formulations that are dynamic and customized to each developmental stage, possibly using bioengineered substrates and time-controlled factor release systems.

### Negative outcomes of IVP: the need for more natural conditions

**Figure 1:**
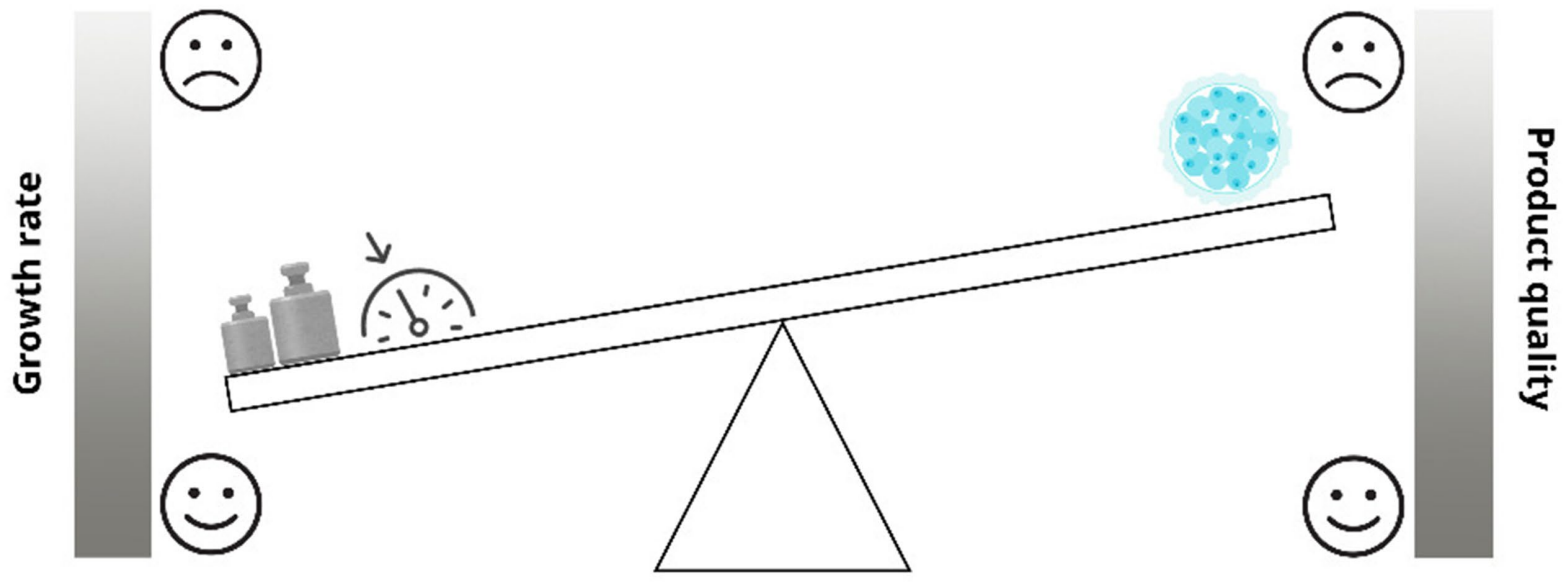
Impact of accelerated *in vitro* conditions on embryo quality: a developmental trade-off. As shown, during the IVP there is the need of a continuous equilibrium, if developmental speed is prioritized the embryo’s quality will be affected.

Despite technological advancements, IVP techniques such as ovum pick-up (OPU) and intracytoplasmic sperm injection (ICSI) still struggle to match natural fertilization success rates (18–21). Common negative outcomes include large offspring syndrome (LOS), which is characterized by macrosomia, dystocia, prolonged gestation, and congenital abnormalities and is due to perturbation of the environment the embryo has to face with (18, 22, 23). One of the main known environmental disruptors is serum, accelerating embryo development and increasing the number of blastocysts produced but reducing their quality (23–25). Metabolic dysregulation is another concern, leading to altered glucose metabolism, increased systolic blood pressure, and higher adiposity (26, 27). Epigenetic alterations are also observed, causing deviations in gene expression that affect long-term health (22, 28). Bovine LOS, analogous to Beckwith-Wiedemann syndrome in humans, underscores the importance of optimizing IVP conditions to minimize epigenetic disruptions (17, 28). During the IVP there is the need of a continuous equilibrium, if developmental speed is prioritized the embryo’s quality will be affected (Figure 1). In future research, reducing reliance on serum-based media and developing dynamic, stage-specific alternatives may improve outcomes.

### Mimicking *in vivo* conditions: the role of reproductive fluids and organoids

To enhance *in vitro* production (IVP) outcomes, researchers are exploring the addition of reproductive fluids to IVP media to improve embryo viability and reduce aberrant growth patterns (29). Mimicking natural conditions in *in vitro* cultured systems is a key factor to obtain high-quality embryos. This can be achieved by supplementing the culture media with different nutritive factors (such as proteins) or by co-culturing embryos with oviductal epithelial cells, which has demonstrated beneficial effects on embryo development (30).

To better resemble the natural environment of the embryo, reproductive fluids (RFs), such as oviductal fluid (OF) and uterine fluid (UF), have been added to *in vitro* systems. At low concentrations, RFs support embryo development and improve quality: OF has been shown to enhance embryo cryotolerance and upregulate the expression of development-related genes, while UF exhibits antioxidant properties (24, 31, 32). However, higher concentrations may exert dose-dependent adverse effects.

RF supplementation aims to reproduce the complex maternal-conceptus communication that occurs *in vivo*, which involves multiple “omics” pathways, paracrine/autocrine signals, and stage-specific molecular cues. These interactions are dynamically influenced by the presence of gametes and embryos and evolve throughout development (33–35). Therefore, the culture medium must be tailored to match the changing needs of the developing embryo.

Recent attention has also focused on extracellular vesicles (EVs) found within RFs. EVs differ between oviductal regions and act as key messengers in embryo–maternal communication. Studies suggest that supplementing *in vitro* culture systems with OF-derived EVs can enhance embryo quality and/or development by influencing, among other, gene expression and lipid metabolism (25, 36).

Additionally, mimicking natural mechanical stimulation in the oviduct is gaining interest. Innovative culture systems that simulate physical forces using tilting platforms, microfluidics, vibration systems, or soft substrate materials have shown promise in replicating the biomechanical environment of the oviduct (35).

A summary of these approaches is presented in Table 1.

**Table 1:** Comparative evaluation of biomimetic strategies for enhancing IVP outcomes.

Approach	Advantages	Limitations
RF Supplementation	Enhances cryotolerance, gene expression	Dose-dependent adverse effects
EV Addition	Supports communication, improves quality	Media-dependent efficacy
Microfluidics	Replicates mechanical cues	Technically complex

### Advancements in 3D culture systems

Unlike 2D cultures, which rapidly lose cellular differentiation and function, 3D cultures maintain essential characteristics such as polarity, ciliary activity, and secretory function resembling *in vivo* conditions (9). Embryonic development is enhanced with improved blastocyst formation rates and a more favorable inner cell mass to trophectoderm ratio (37). Organoids are composed of key components, including adult stem cells (ASCs) or induced pluripotent stem cells (iPSCs), an extracellular matrix (ECM) that provides structural support, and growth factors that maintain cellular differentiation and function (38). Future directions in this field include 3D bioprinting to recreate oviductal microstructures and the use of microfluidic systems to simulate dynamic hormonal and gamete interactions (39). For instance, Belda-Perez et al. (37) demonstrated that 3D-printed culture materials can improve the performance and biocompatibility of *in vitro* embryo development systems.

### Challenges and limitations

Current models cannot fully mimic complex parameters such as sperm selection by uterine-tubal junction (UTJ), time-dependent EV changes, or dynamic hormonal fluctuations. Ethical and biosafety implications are underdeveloped: potential risks include immunological reactions and regulatory concerns related to using biological fluids or stem-cell derived structures *in vitro*.

### Translational models

A table summarizing the current advances per species (see Table 2) has been provided to highlight translational opportunities. Ethical frameworks and cost–benefit analyses will be essential to transition these innovations from laboratory settings to routine clinical or production applications.

**Table 2:** Species-specific advances.

Study reference	Species	Technique/model	Key finding
París-Oller et al. (17)	Piglets	RF Supplementation	Reduced IVP–AI differences
Guidobaldi et al. (14)	Rabbits	Cumulus-derived chemoattractants	Sperm guidance via progesterone
Lopera-Vasquez et al. (25, 31)	Cows	EV supplementation	Improved embryo quality
Belda-Perez et al. (37)	Cows	3D printing	Improved culture performance
Cui et al. (38)	Humans	Organoid development	Disease modeling potential

### Integration with artificial intelligence (AI)

Many types of AI models have been proposed for the optimization of *in vitro* embryo productions. Despite the undoubtable value of the algorithms created to reduce the subjectivity of embryo assessment, shortening the training curve for new embryologists, and improving consistency across practitioners not yet shared AI models are available. To validate the performance of an AI model in a clinical context and to reveal any improvements over current practices are necessary randomized controlled trials (40–42).

## Conclusion

Recreating the oviductal microenvironment in *in vitro* production (IVP) systems represents a major advancement in reproductive biotechnology. However, calling it a “paradigm shift” requires further substantiation—perhaps in reference to changing how future embryo technologies are conceptualized and regulated. To bring assisted reproduction closer to its natural counterpart, future research should prioritize the standardization of reproductive fluid supplementation (25), scalable organoid systems (38), and the validation of microfluidic technologies under field conditions (17, 37).

To date, the integration of biophysical cues, molecular signals, and cellular architecture in IVP remains fragmented. Coordinated multi-disciplinary efforts are needed to create fully biomimetic systems that reflect the temporal and spatial complexity of the oviductal environment.

In addition, creating species-specific protocols and cross-comparison models using data from bovine, porcine, rabbit, and human studies will be critical to fine-tuning these technologies.
